# Oral microbial communities in 5-year-old children with *versus* without dental caries

**DOI:** 10.1186/s12903-023-03055-2

**Published:** 2023-06-16

**Authors:** Zhengyan Yang, Ting Cai, Yueheng Li, Dan Jiang, Jun Luo, Zhi Zhou

**Affiliations:** 1 Stomatological Hospital of Chongqing Medical University, Chongqing, 400015 China; 2 Chongqing Key Laboratory of Oral Biomedical Engineering of Higher Education, Chongqing, 400015 China; 3 Chongqing Key Laboratory of Oral Diseases and Biomedical Sciences, Chongqing, 400015 China

**Keywords:** Microbial community, Preschool children, Dental caries, High-throughput sequencing, Saliva, Biodiversity

## Abstract

**Background:**

Caries in young children has received more and more attention. The study of the oral microbiota may help to understand the polymicrobial etiology of dental caries.

**Objectives:**

To investigate the diversity and structure of microbial communities in saliva samples from 5-year-old children with versus without dental caries.

**Methods:**

A total of 36 saliva samples were collected from 18 children with high caries (HB group) and from 18 children without caries (NB group). Then, 16S rDNA was amplified from bacterial samples using polymerase chain reaction, and high-throughput sequencing was performed using Illumina Novaseq platforms.

**Results:**

Sequences were clustered into operational taxonomic units (OTUs), which were distributed among 16 phyla, 26 classes, 56 orders, 93 families, 173 genera, and 218 species. *Firmicutes, Bacteroides, Proteobacteria, Actinobacteria, Fusobacteria*, *Patescibacteria, Epsilonbacteraeota, Cyanobacteria, Acidobacteria* and *Spirochaetes* were basically the same in different groups, but their relative abundances were different. The core microbiome was defined as the species from 218 shared microbial taxa. The alpha diversity test showed that there were no significant differences in microbial abundance and diversity between the high caries and no caries groups. The results from principal coordinate analysis (PCoA) and hierarchical clustering showed that the two groups had similar microorganisms. The biomarkers of different groups were defined by LEfSe analysis to identify potential caries-related and health-related bacteria. Co-occurrence network analysis of dominant genera showed that oral microbial communities in the no caries group were more complex and aggregated than those in the high caries group. Finally, the PICRUSt algorithm was used to predict the function of the microbial communities from saliva samples. The obtained results showed that mineral absorption was greater in the no caries group than in the high caries group. BugBase was used to determine phenotypes present in microbial community samples. The obtained results showed that *Streptococcus* was greater in the high caries group than in the no caries group.

**Conclusion:**

Findings of this study provide a comprehensive understanding of the microbiological etiology of dental caries in 5-year-old children and are expected to provide new methods for its prevention and treatment.

**Supplementary Information:**

The online version contains supplementary material available at 10.1186/s12903-023-03055-2.

## Introduction

It is well known that by the age of 5 years, primary dentition is fully established and relatively stable. The 4^th^ National Oral Health Survey in mainland China in 2018 [1] revealed that in the past decade, the prevalence of dental caries in young children in China was not optimistic, and the prevalence rate showed an upward trend. The prevalence rate of dental caries in 5-year-old children was 71.9%, which was 5.8% higher than that 10 years ago. The average number of caries per child was 4.24, but the number of teeth filled due to caries was only 4.1%. Currently, the etiology of dental caries that has been accepted by most scholars is the theory of four main factors, in which bacteria play a crucial role in the initiation of dental caries.

Dental caries is defined as dysbiosis rather than a chronic infectious disease. In 1994, Marsh [2] proposed the ecological plaque hypothesis. They found that due to changes in the oral environment, such as changes in sugar intake, diet or pH value, the relatively balanced bacterial composition in the biofilm can change remarkably, which shifts the microbiome toward cariogenic bacteria. For example, in a low pH environment, the proportion of potential cariogenic bacteria such as *Streptococcus mutans* and *Lactobacilli* will increase, which may break the ecological balance of the oral microbial community, leading to dental caries. This hypothesis proposes that both microorganisms and the environment are factors that induce dental caries [3]. All found cariogenic bacteria are normal flora in the mouth, which are interdependent and maintain a dynamic balance. When the environment changes, the ecological balance of microorganisms in dental plaque, tooth surface and environment is broken, causing dental caries. Dr. Marsh proposed that dental caries can be prevented not only by targeting cariogenic bacteria but also by interfering with the process of breaking the ecological balance. Takahashi et al. [4] proposed that the cariogenic microenvironment is a microenvironment that transitions from the dynamic stability stage to the acid production stage and finally develops to the acid resistance stage. The occurrence and development of dental caries result from an imbalance in the microbial community.

Strong evidence exists that during the development of healthy individuals, oral microbial communities change dynamically, and the oral microbiome changes physiologically. Microbial composition is specific in different age groups. In addition to *Streptococcus mutans*, many types of bacteria, such as *Veillonellales, Actinomyces, Granulosicoccus, Leptotrichia, Thiomonas, Bifidobacterium and Prevotella,* are closely related to dental caries. The imbalance of oral microbial communities may be an important mechanism of dental caries. In this study, we performed Illumina Novaseq sequencing to investigate the diversity and structure of microbial communities in saliva samples from 5-year-old children with versus without dental caries. Findings from this study will help understand the microbial etiology of dental caries in 5-year-old children and provide evidence for developing novel methods to prevent and treat dental caries.

## Materials and methods

### Subject selection

Patients with high caries and no caries were recruited from the same kindergarten in Yubei District of Chongqing, China. The inclusion criteria of subjects are as follows: (i) ages 54 to 66 months; (ii) no upper respiratory infections in the past fortnight; (iii) no antibiotic use in the past 2 months; (iv) no partial denture and appliance, and (v) no history of systemic diseases, oral diseases and dental treatments. Eighteen high caries (decayed, missing and filled teeth (dmft) index ≥ 6) participants and 18 no caries (dmft = 0) participants were finally selected. The definitions and diagnoses of dental caries are based on the criteria of the World Health Organization [5]. All participants underwent a comprehensive oral examination from a specialized dentist. These participants’ parents or grandparents were sufficiently informed about the aims of the research and signed the written informed consent provided by the Ethics Committee of the Stomatological Hospital of Chongqing Medical University.

### Sample collection

Before the samples of all participants were taken, sterile water was used to rinse their mouths, and they were not allowed to eat, drink or brush their teeth for 2 h. A specialized dentist collected their unstimulated saliva, placed it into 1.5-mL sterile microcentrifuge tubes, and froze it at -80 °C for further use.

### DNA extraction, PCR amplification and illumina novaseq sequencing

Based on the manufacturer’s protocol, microbial DNA was extracted from all specimens by the PowerSoil® DNA Isolation Kit. Primers 338F (5′-ACTCCTACGGGAGGCAGCAG-3′) and 806R (5′-GGACTACHVGGGTWTCTAAT-3′) were used to amplify the V3-V4 hypervariable regions of the bacterial 16S rRNA gene using PCR on the Veriti 96-Well Thermal Cycler (GeneAmp 9902, ABI, USA). Target area PCR was conducted with the following program: initial denaturation at 98 °C for 2 min, 30 cycles of denaturation at 98 °C for 30 s, annealing at 50 °C for 30 s, elongation at 72 °C for 60 s, and a final extension at 72 °C for 5 min, followed by storage at 4 °C. Target area PCR was performed in triplicate with a 30 µL mixture containing 15 µL of KOD FX Neo Buffer, 6 µL of dNTPs (2 mM each), 0.9 µL of Vn F (10 µM)/Vn R (10 μM), 0.6 µL of KOD FX Neo and 50 ng ± 20% of template DNA. Solexa PCR was conducted with the following program: initial denaturation at 98 °C for 30 s, 10 cycles of denaturation at 98 °C for 10 s, annealing at 65 °C for 30 s, elongation at 72 °C for 60 s, and a final extension at 72 °C for 5 min.

Solexa PCR was performed in triplicate with a 20-µL mixture containing 5 µL of target area PCR products, 10 µL of 2 × Q5 HF MM, and 2.5 µL of MPPI-a (2 µM)/MPPI-b (2 μM).

The final PCR products were extracted from a 1.8% agarose gel under a voltage of 120 V. Forty minutes later, overcolumn purification was performed using an OMEGA DNA purification column. The PCR products were purified, quantified, and homogenized to form a sequencing library. The built libraries were first subjected to library quality inspection, and the qualified libraries were sequenced by Illumina NovaSeq 6000 PE250.

### Processing of sequencing data [6]

Primary FastQ files were divided into multiple files for processing and quality-filtered by Trimmomatic [7]. The following standards were used for sequence combination. (i) The reads were cut off when meeting any site with an average quality score < 20 over a 50 bp sliding window. (ii) The primers were perfectly matched, allowing the mismatch of two nucleotides, and reads with ambiguous bases were not allowed. (iii) Sequences with overlap longer than 10 bp were merged based on their overlap sequence.

Using USEARCH [8] (version 10.0), operational taxonomic units (OTUs) were clustered with a 97% similarity cut off. Additionally, chimeric sequences were recognized and deleted by UCHIME (version 8.1) [9]. By comparing the RDP Classifier algorithm [10] (version 2.2, http://sourceforge.net/projects/rdpclassifier/) against the Silva [11] 16S rRNA (Release128, http://www.arb-silva.de) database, the taxonomy of each 16S rRNA gene sequence was analyzed based on an 80% confidence threshold.

### Bioinformatics and statistical analysis [6]

Bioinformatics analysis was conducted using QIIME. The alpha diversity indices of Shannon, Simpson, Chao, ACE and PD_whole_tree were calculated at 97% identity by Mothur [12] version v.1.30. Beta diversity analysis was performed by principal coordinate analysis (PCoA) based on Bray–Curtis distances at the OTU level. A hierarchical clustering analysis based on weighted UniFrac distances was also conducted [13]. Analysis of similarities (ANOSIM) was performed using the vegan package in the R language and drawn with Python; unweighted UniFrac distances were used to compare different groups. Welch’s *t*-test was used to compare the relative abundance of predominant bacteria between different groups. A Venn diagram was used to define the core microbiome at the species level using Mothur [12]. Linear discriminant analysis of effect size (LEfSe) was conducted to define the biomarkers of the two groups. The threshold on the logarithmic LDA score for distinguishing features was set to 3 [14]. Co-occurrence analysis among the genera was performed with Python, and co-occurrence analysis of the 80 richest genera of each group was performed at the same time. The phylogenetic investigation of communities by reconstruction of unobserved states (PICRUSt2) program was used to predict 16S rRNA-based data from high-throughput sequencing and to further analyze the composition and differential of the Kyoto Encyclopedia of Genes and Genomes (KEGG) [15] metabolic pathway in the context of IMG microbial genome data. BugBase normalizes the OTU by the predicted 16S copy number and then predicts the microbial phenotype [16] using the provided precomputed files. Differences were considered significant when *P* < 0.05 and extremely significant when *P* < 0.01. SPSS 25.0 software (SPSS Inc., Chicago, IL, USA) was used for statistical analysis. The raw data will be made available by the authors without undue reservation to any qualified researcher.

## Results

### Sequence information

**Fig. 1:**
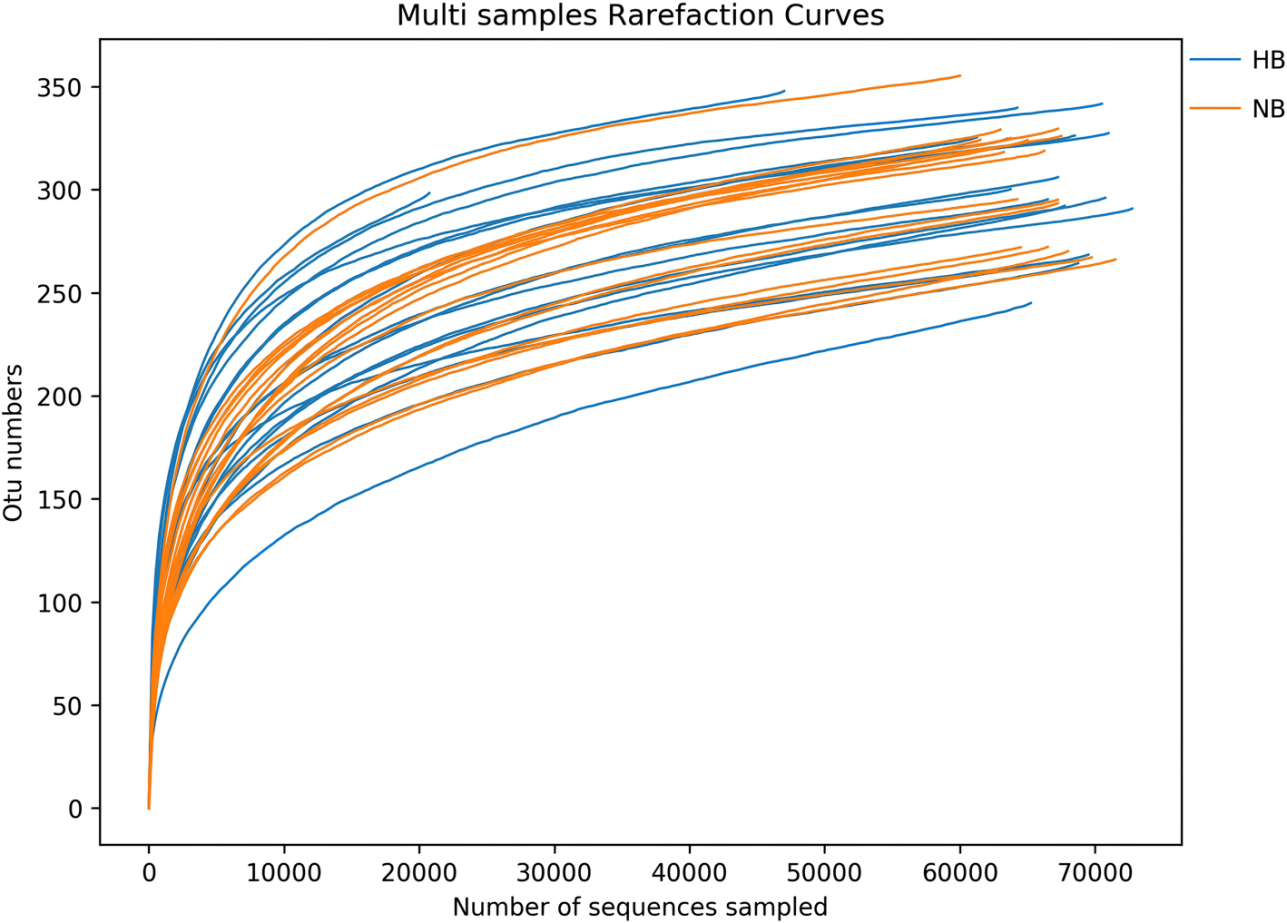
The rarefaction curves at the genus level. ^✳^The x-axis indicates the number of sequences sampled. The y-axis indicates the number of operational taxonomic units (OTUs). Different colors represent different groups

We collected saliva samples from 36 children with an average age of 60.2 months. All saliva samples were divided into two groups: the no caries (NB) group (*n* = 18) and the high caries (HB) group (*n* = 18). After Illumina NovaSeq sequencing, 2,525,425 effective sequences were acquired from 36 saliva samples, with an average of 70,150 sequences per sample. The average length of the sequences was 423 bp. Ninety-seven percent of qualified sequences were clustered, and 10980 (Appendix 1) operational taxonomic units (OTUs) were obtained. Good's coverage estimation values of > 99.9% and the rarefaction curves (Fig. 1) reaching the even stage indicated that sampling was basically completed.

### Alpha diversity

The alpha diversity of Shannon, Simpson, Chao, ACE and PD_ whole_tree was calculated to analyze the diversity and abundance of all samples. Student’s t-test was performed to compare the saliva samples between the high caries and no caries groups. There were no significant differences in alpha diversity indices between the high caries and no caries groups. This suggests that the diversity and abundance of oral microbial communities were similar between the high caries and no caries groups.

### Microbial community structure

**Fig. 2:**
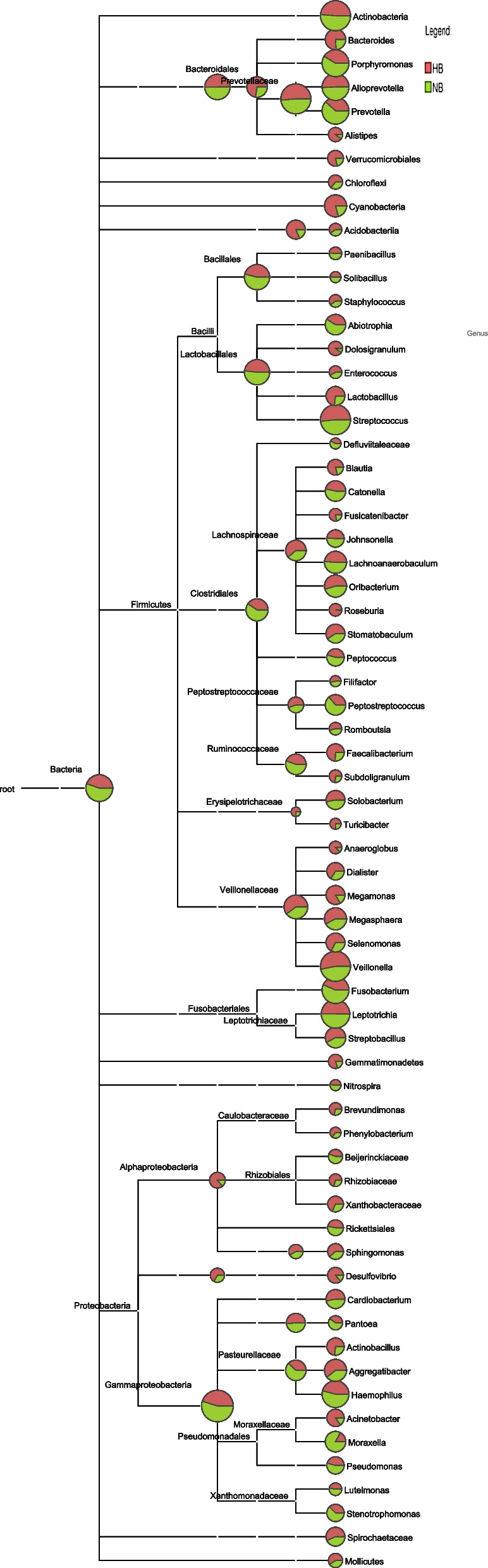
The phylogenetic tree of the 68 most abundant genera. Each branch represents a taxon, the length shows phylogenic distances between two taxa, and different colors represent different phyla. The bar plot on the right side shows the relative abundance of each genus in the high caries and no caries groups

**Fig. 3:**
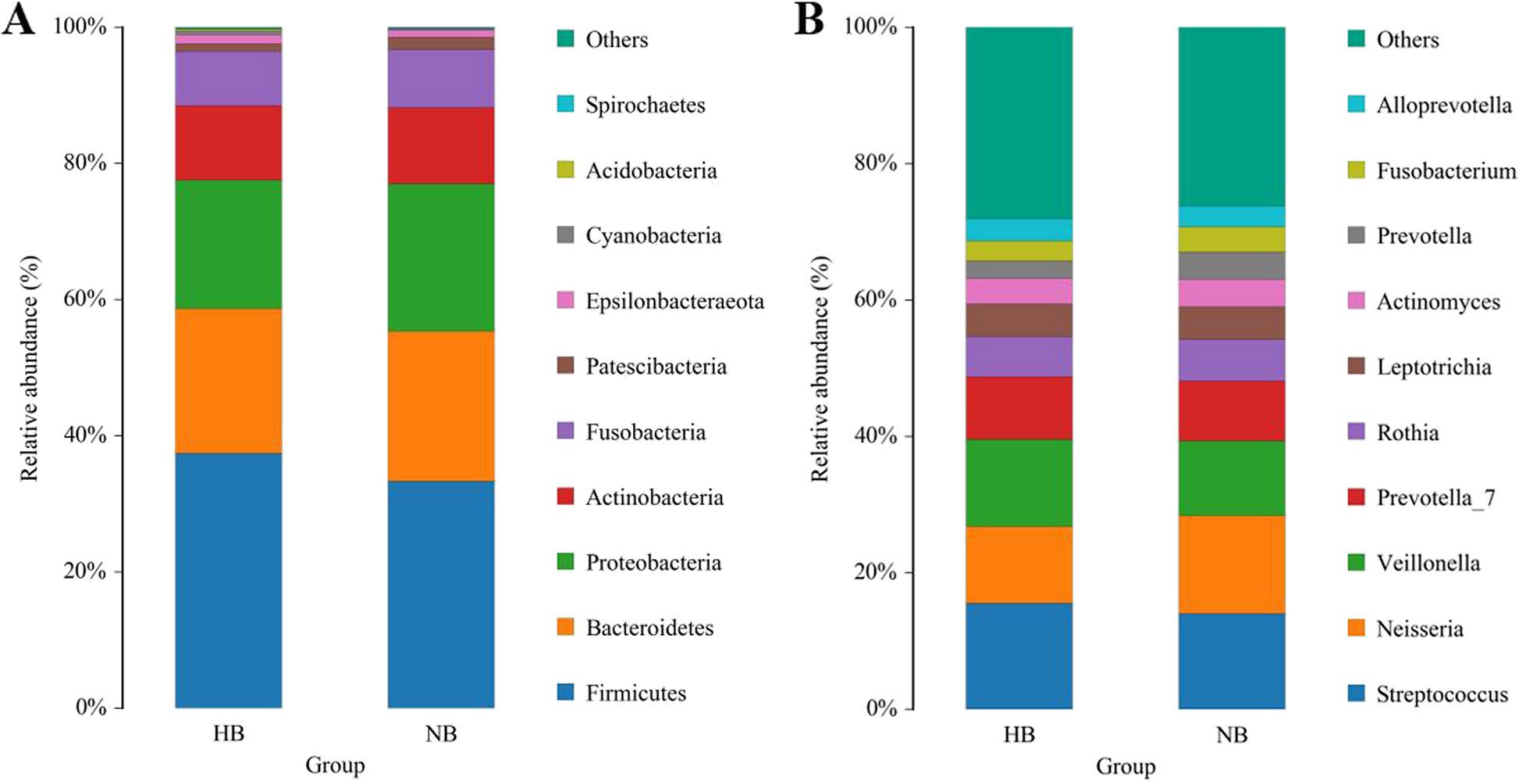
The distribution of the predominant bacteria. **A** Results at the phylum level. **B** Results at the genus level. The predominant taxa (relative abundance > 2% on average) are shown

**Fig. 4:**
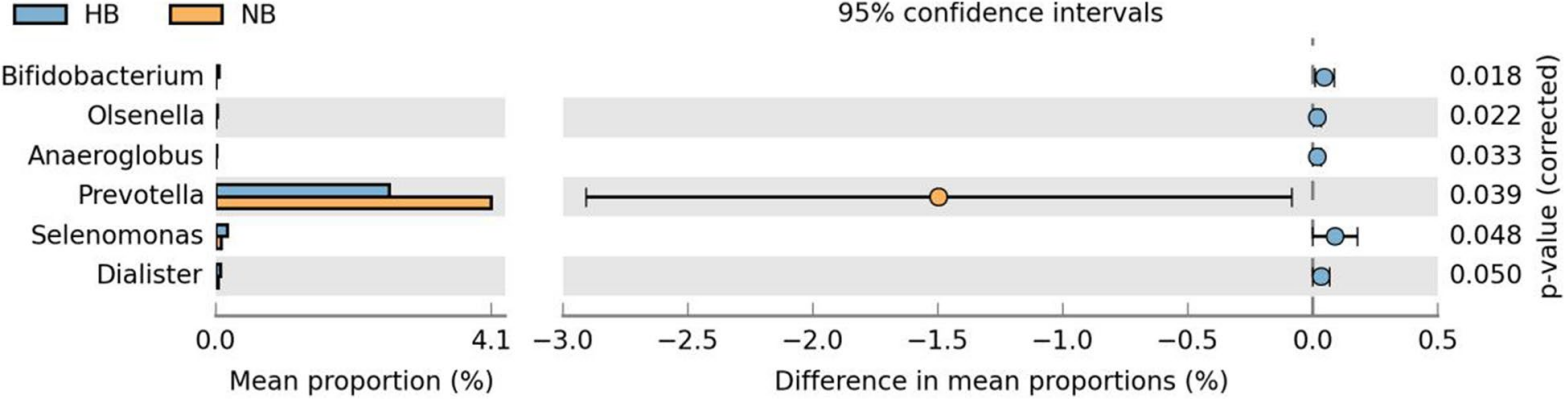
Welch’s t-test bar plot at the genus level. The results of the high caries and no caries groups. Only the results that had significant differences are shown (*P* < 0.05)

**Fig. 5:**
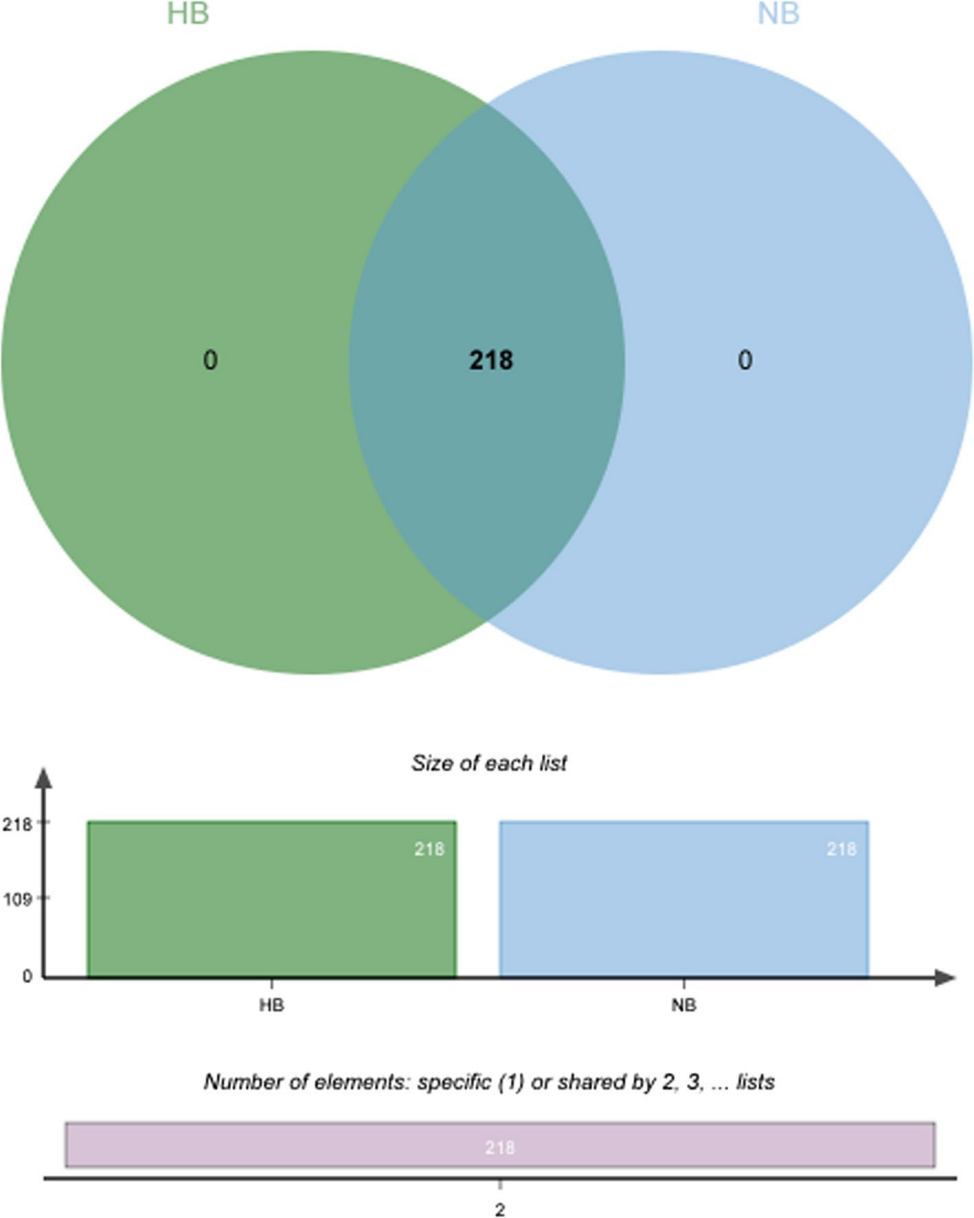
Venn diagram at the species level. Different colors represent different groups. The overlaps represent the common species between groups, and the nonoverlapping portions represent unique species in each group

OTUs were distributed among 16 phyla, 26 classes, 56 orders, 93 families, 173 genera, and 218 species. The phylogenetic trees of the 68 most abundant genera were constructed, in which the taxonomic composition and abundance can be observed (Fig. 2). The most abundant phyla were *Firmicutes* (37.4%), *Bacteroidetes* (21.3%), *Proteobacteria* (18.8%), *Actinobacteria* (10.9%), *Fusobacteria* (8.0%), *Patescibacteria* (1.1%), *Epsilonbacteraeota* (1.3%), *Cyanobacteria* (0.6%), *Acidobacteria* (0.3%) and *Spirochaetes* (0.2%), all of which accounted for 99.8% of the total sequences. The most abundant genera were *Streptococcus* (15.5%), *Neisseria* (11.2%), *Veillonella* (12.7%), *Prevotella_7* (9.1%), *Rothia* (5.9%), *Leptotrichia* (4.9%), *Actinomyces* (3.7%), *Prevotella* (2.6%), *Fusobacterium* (2.9%), and *Alloprevotella* (3.3%), all of which accounted for 71.9% of the total (Fig. 3). The predominant bacteria between the two groups were largely consistent, but their relative abundances were different. The relative abundances of *Selenomonas*, *Bifidobacterium*, *Dialister*, *Olsenella*, and *Anaeroglobus* were significantly higher in the high caries group than in the no caries group. The relative abundance of *Prevotella* was significantly lower in the high caries group than in the no caries group (Fig. 4). The core microbiome was defined by a Venn diagram, which can be detected in most individuals at the species level. We identified 218 species in each group (Fig. 5). Among them, all species were consistent, indicating that the composition of microorganisms was stable in the saliva of the high caries and no caries groups.

### Similarity and dissimilarity of bacterial compositions

**Fig. 6:**
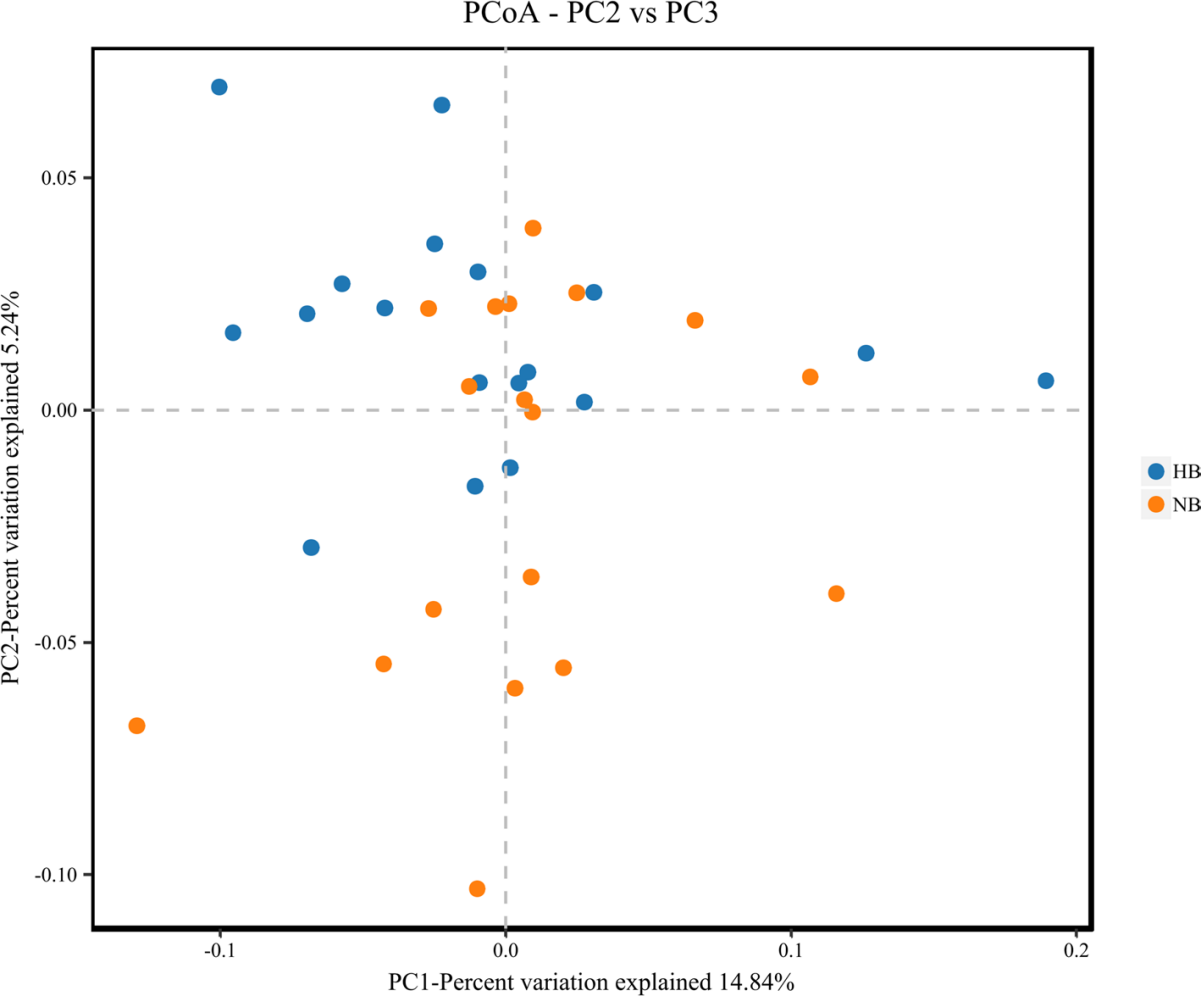
PCoA based on Bray–Curtis distances at the OTU level at 97% identity. Each sample is represented by a dot. Circles in different colors represent different groups. PC1 explained 14.84% of the variation observed, and PC2 explained 5.24% of the variation

**Fig. 7:**
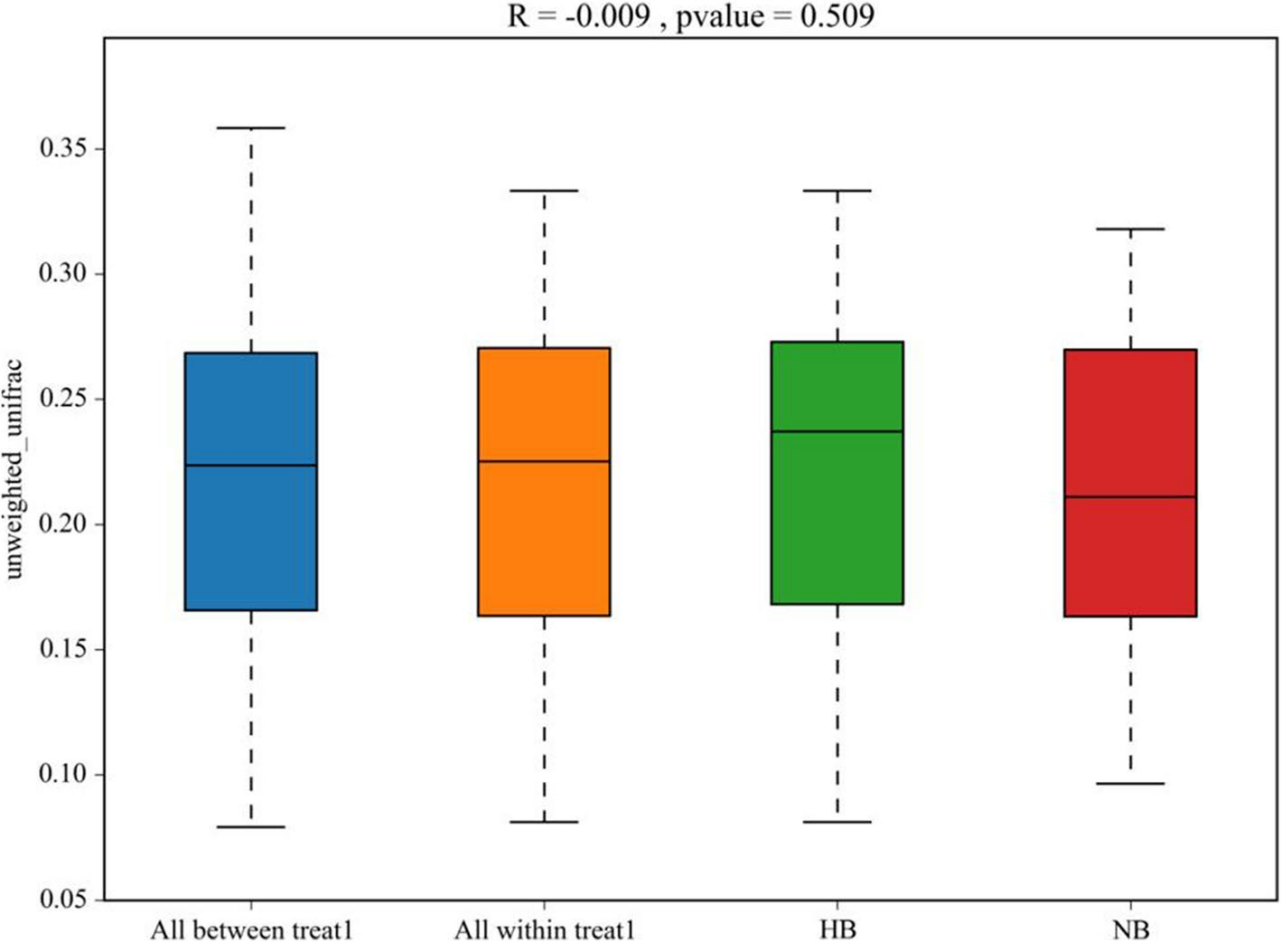
ANOSIM based on unweighted UniFrac distances between high caries and no caries groups (*P* = 0.509)

**Fig. 8:**
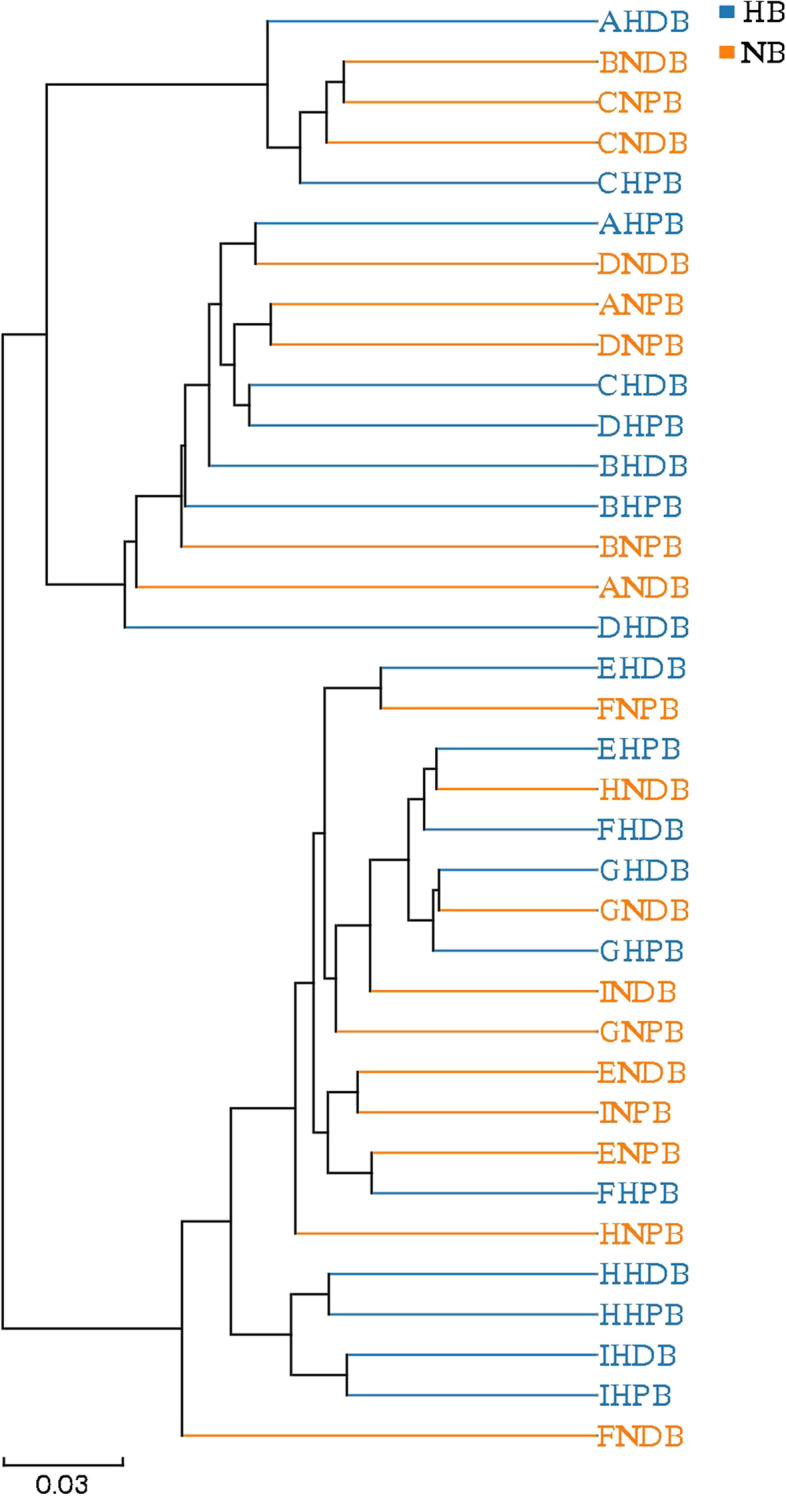
Hierarchical clustering tree at the OTU level. The length of the branch shows the distance between two samples, and different groups are presented in different colors

PCoA was used to evaluate the similarity of the microbial community structure between the two groups (Fig. 6). PCoA results showed that there was no significant difference in bacterial composition between the high caries and no caries groups, suggesting that patients with high caries and no caries had similar microbial community structures. The ANOSIM test (Fig. 7) and hierarchical cluster analysis (Fig. 8) also showed no remarkable difference between high caries and no caries samples.

**Fig. 9:**
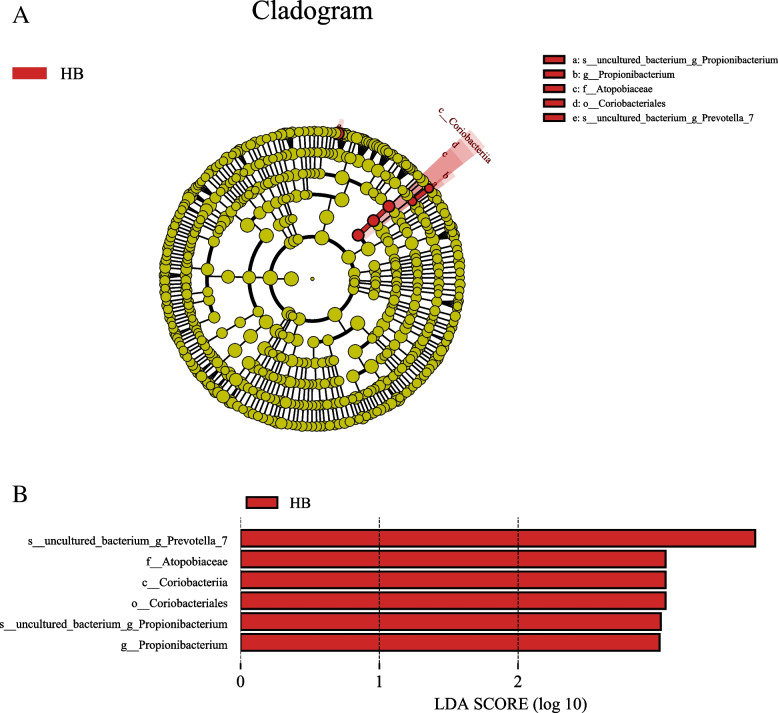
The potential biomarkers were defined by LEfSe. **A** Cladogram for taxonomic representation of significant differences between high caries and no caries groups. The colored nodes from the inner to the outer circles represent taxa from the phylum to genus level. The significantly different taxa are marked by different colors representing the four groups. **B** Histogram of the LDA scores for differentially abundant features among groups. The threshold on the logarithmic LDA score for discriminative features was set to 3.0

The features most likely to explain differences between high caries and no caries samples were defined by LEfSe. Figure 9 shows a branching diagram of potential biomarkers representing different groups. At the genus level, *Propionibacterium* was remarkably enriched in the high caries group. At the species level, the abundances of *s_uncultured_bacterium_g_Prevotella_7* and *s_uncultured_bacterium_g_Propionibacterium* were relatively higher in the high caries group than in the no caries group (LEfSe LDA = 3, *P* < 0.05).

### Network analysis and function prediction

**Fig. 10:**
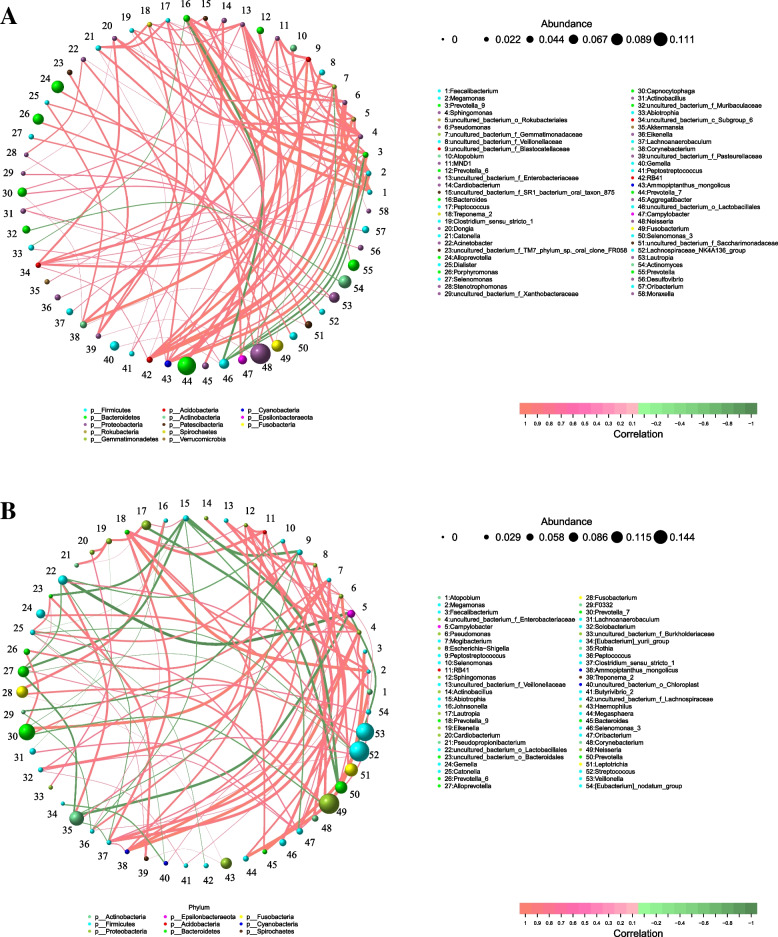
Network analysis showed interactions of genera (|SpearmanCoef|> 0.8 and *P* < 0.01). Bacterial interactions of two different groups. The size of the node is proportional to the genera abundance. Node color corresponds to phylum taxonomic classification. Edge color represents positive (red) and negative (green) correlations

Co-occurrence analysis was performed to identify the interactions between genera in different groups. The top 80 genera with high relative abundance were found to have complex interactions in each group. In the high caries group, 7 genera exhibited negative correlations (Fig. 10A), but in the no caries group, 27 genera exhibited negative correlations (Fig. 10B). Microbial communities were more negatively correlated and showed a more complex relationship in the no caries group than in the high caries group.

**Fig. 11:**
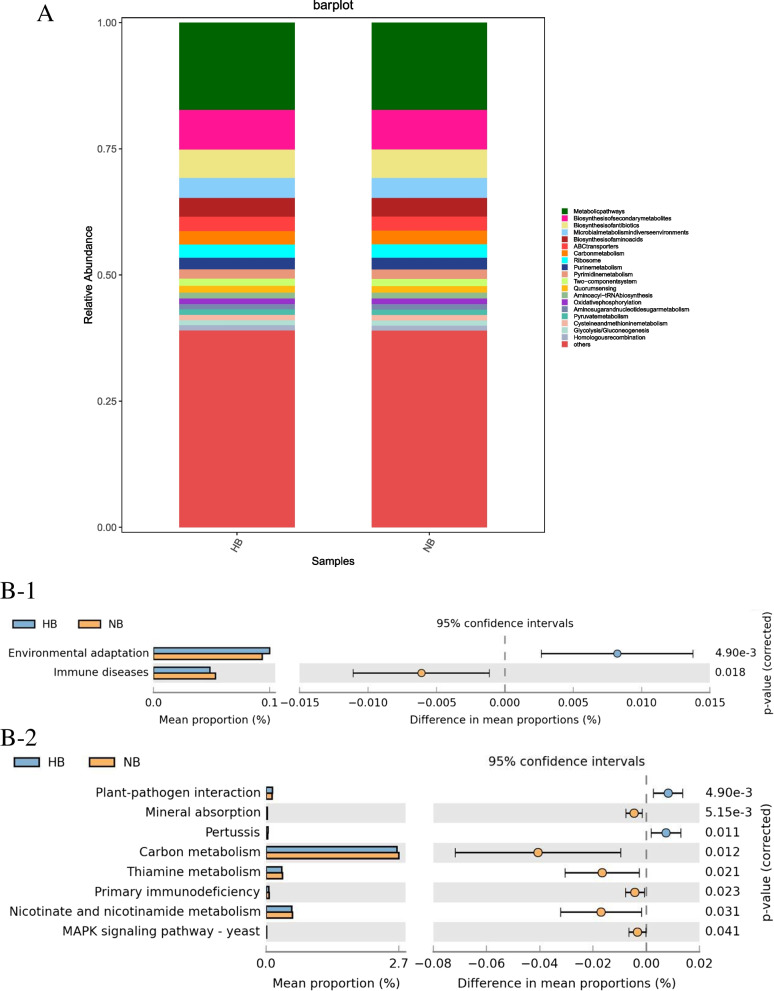
Function prediction by PICRUSt. **A** The compositions of KEGG functions in the two groups. **B** Welch’s t-test bar plot of KEGG. **B1** Results of the no caries and high caries groups in Class 2. **B2** Results of the high caries and no caries groups in Class 3. Only the results that had significant differences are shown (*P* < 0.05)

PICRUSt2 was used to help understand the function of microbial communities in saliva samples. A bar graph (Fig. 11A) showed that the saliva samples of the two groups had similar KEGG maps, suggesting that the function of microbiota in the two groups was similar. However, STAMP analysis showed that there was a difference in the function of microbiota between the two groups. In class 2 (Fig. 11B-1), a higher abundance of environmental adaptation was observed in the high caries group than in the no caries group and a higher abundance of immune diseases was observed in the no cariesgroup than in the high caries group (*P* < 0.05). In class 3 (Fig. 11B-2), a higher abundance of plant-pathogen interaction and pertussis were observed in the high caries group than in the no caries group, while a higher abundance of mineral absorption, carbon metabolism, thiamine metabolism, primary immunodeficiency, nicotinate and nicotinamide metabolism, and MAPK signaling pathway-yeast, were observed in the no caries group than in the high caries group (all *P* < 0.05).

**Fig. 12:**
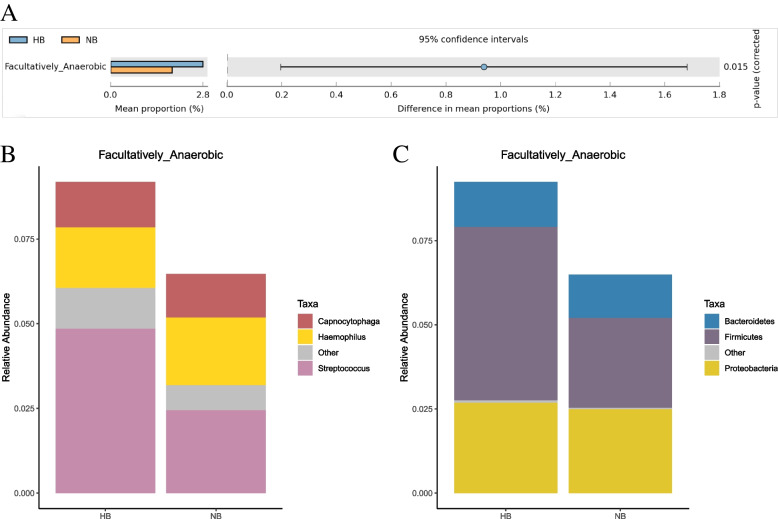
BugBase phenotype prediction. **A** Welch’s t-test bar plot of phenotype in the two groups (red represents a significant difference (*P* < 0.05)). **B** The compositions of different phenotypes at the genus level. **C** The compositions of different phenotypes at the phylum level

BugBase was used to determine phenotypes present in microbiota samples. Nine phenotypes including Aerobic, Anaerobic, Contains_Mobile_Elements, Facultatively_Anaerobic, Forms_Biofilms, Gram_Negative, Gram_Positive, Potentially_Pathogenic, Stress_Tolerant were predicted from both groups. The relative abundance of facultative anaerobic was significantly higher in the high caries group than in the no caries group (*P* < 0.05) (Fig. 12A). At the genus level, *Streptococcus* caused a higher relative abundance of facultative anaerobic bacteria in the high caries group than in the no caries group (Fig. 12B). At the phylum level, *Firmicutes* caused a higher relative abundance of facultative anaerobic bacteria in the high caries group than in the no caries group (Fig. 12C).

## Discussion

Bacteria in dental biofilms are considered to be essential in the occurrence and development of dental caries [2, 17]. To comprehensively study the oral microbiome and understand its etiology, high-throughput technology is an effective method to study the composition and structure of microbial communities related to health and disease.

Previous studies have helped us to increase our understanding of microbial compositions at different ages. Some researchers found that *Firmicutes* was lower and *Proteobacteria* was higher in children than in adolescents and adults, while the conclusions were completely opposite in adolescents and adults [18]. According to a previous study [19], it is generally believed that microorganisms attached to the surface of teeth and mucous membranes continue to flow into saliva, making it the repository of oral microbial communities and a "fingerprint" of all oral microbiota. Findings of this study in which saliva samples were used will help us to comprehensively understand the microbial communities associated with early childhood dental caries.

Previous studies have reported that when dental caries occurs, the alpha diversity of microbial communities in the mouth will decrease [20, 21]. In contrast, the alpha diversity of microbial communities measured in this study demonstrated that the diversity and abundance of microbial communities were similar between the high caries and no caries groups. This is also supported by the results of a previous study [22]. It is speculated that the reason for the inconsistent results between different studies may be attributed to a certain extent to the choice of subjects. The subjects in this study have high homogeneity. All subjects lived in the same area and had similar diets and lifestyles. Although their salivary microbial communities have certain differences in species composition, their microbial diversities are highly similar.

The detected OTUs were distributed among 16 phyla, 26 classes, 56 orders, 93 families, 173 genera, and 218 species. Phyla mainly included *Firmicutes, Bacteroides, Proteobacteria, Actinobacteria, Fusobacteria*, *Patescibacteria, Epsilonbacteraeota, Cyanobacteria, Acidobacteria* and *Spirochaetes.* These results are similar to previous findings [23, 24]. This indicates that the microbial community structure in the mouth is relatively stable. It can be seen from the community bar graph that the microbial community structure in the high caries group was similar to that in the no caries group. This indicates that the disease state may not have a remarkable impact on bacterial composition.

At the genus level, the most popular bacteria in the two groups were basically the same, but the relative abundances were different. *Prevotella* is often detected in the study of caries-related microorganisms in children, and *Prevotella* is considered to be used as a marker to detect caries in young children [25]. *Prevotella* is a gram-negative bacterium of the *Bacteroidetes phylum* that has been considered a potential periodontal pathogen for a long time. Its role in dental caries has been recognized in a recent study [26]. However, in this study, *Prevotella* was higher in the no caries group than in the high caries group. This finding supports the "ecological plaque hypothesis", which states that dental caries is the result of the destruction of the steady state of resident microorganisms rather than the activity of specific microorganisms [27].

LEfSe shows different groups of potential biomarkers. Among these remarkably different genera, *Propionibacterium* showed higher abundance in the high caries group than in the no caries group. Xiao et al. [28] isolated *Propionibacterium* from human caries. They found that the main terminal metabolites of *Propionibacterium* are a large amount of propionic acid and acetic acid, as well as an appropriate amount of succinic acid. *Streptococcus mutans* is a type of *Streptococcus* that is recognized as one of the main cariogenic bacteria; however, in this study, no differences in *Streptococcus mutans* were found between the high caries and no caries groups. Agnello et al. [29] found that the oral microbial communities in Aboriginal children with severe early childhood caries were remarkably different from those in children with no caries. In addition, they detected extremely abundant *Streptococcus mutans* in the microbial plaque of children with severe early childhood caries. However, due to different population characteristics and analysis methods, some scholars also found that the relative abundance of *Streptococcus* in the microbial plaque decreased when caries occurred [18]. In many healthy subjects, the content of *Streptococcus mutans* was higher than that detected in patients with white spot lesions and dentin caries lesions [21, 30]. A high abundance of *Streptococcus mutans* does not always exist in the microbial plaque of patients with different degrees of caries [31]. The percentage of high abundance of *Streptococcus mutans* in the microbial community is not linearly correlated with the progress of caries from healthy teeth to deep caries [21]. Therefore, some scholars believe that the level of *Streptococcus mutans* is not significantly correlated with the severity of caries [32]. Epidemiological and caries risk assessment data also show that in recent decades, the use of caries preventive methods taking *Streptococcus mutans* as the main target has had little effect.

Network analysis showed the potential correlation of oral microbiota with dental caries. Different groups show different bacterial interactions. The relationship between oral microbial communities in the no caries group was more complex than that in the high caries group. This finding indicates that some microbial relationships may be destroyed, leading to ecological imbalance and resulting in the development of caries. This is consistent with the finding from a previous study [33].

In this study, we analyzed the function of the salivary microbial community in 5-year-old children from Chongqing, China. We found that when the function of the salivary microbial community was class 3, Mineral absorption gene clusters were concentrated in saliva of children without caries. The possible reason is that the occurrence of dental caries is related to tooth mineralization. The microorganisms in the saliva of children without caries can promote the active absorption of minerals, resist the damage of acid to tooth enamel and the loss of minerals, thus preventing the occurrence of caries. Wang et al. [34] found that the functional difference between children with high caries and no caries was mainly reflected in carbohydrate metabolism. Liu et al. [35] studied the microbial function in deep dentinal carious lesions and found that carbohydrate metabolism and amino acid metabolism drive the occurrence of caries. However, no differences in carbohydrate and amino acid metabolism were found in this study. These findings suggest that in addition to further investigating the products of functional genes of carbohydrate and amino acid metabolism and analyzing the role of carbohydrates in oral flora metabolism, other metabolic pathways, such as mineral absorption, can provide a new entry point for etiology research, prevention and risk assessment of dental caries.

BugBase analysis showed that there was a phenotypic difference in Facultatively_Anaerobic between the high caries and no caries groups. The obtained results also showed that at the genus level, *Streptococcus* was the main factor that caused the increase in Facultatively_Anaerobic in the high caries group. Previous studies have fully revealed the role of *Streptococcus* in the occurrence and development of dental caries [36, 37]. However, there was no significant difference in the content of *Streptococcus* between the high caries and no caries groups. This finding was also supported by the results of other relevant studies [38–40]. It is speculated that this result occurs possibly because *Streptococcus* contains many bacterial species, such as *Streptococcus mutans*, *Streptococcus sanguinis*, *Streptococcus oralis*, *Streptococcus sobrinus*, and *Streptococcus mitis*. These bacterial species play different roles in the occurrence of caries; thus, the relative contents may have different trends in the occurrence and development of dental caries, resulting in the stability of the overall content of *Streptococcus*. The mouth, as part of the digestive system, is located at the beginning of the whole system, and it is in direct contact with the external environment. Therefore, the overall composition of oral microbial communities shows a high degree of dynamics [41]. Temperature, pH, salinity, redox potential, and the acquisition of oxygen or nutrients also have a certain impact on microbes. Under normal conditions, the microbial community present in the mouth can resist the invasion and colonization by alien species; thus, it can regulate local and systemic health. On the other hand, the imbalance of the microbial community may lead to oral diseases such as common dental caries and periodontal disease [42, 43]. According to the "ecological plaque hypothesis", dental caries results from the disruption of the normal microbial community balance due to changes in the oral environment [3]. The results of this study once again show that the microbial changes involved in dental caries are more complex than expected. The occurrence of dental caries is not caused by a specific bacterium, but the structure of microbial communities among all microorganisms in the mouth has changed [3]. The eruption of permanent teeth and the increase in age will affect oral microorganisms [30, 44]; therefore, the study of the oral microbiome should be based on age. To exclude the influence of age, in this study, we selected only 5-year-old children as subjects. To try to control the effect of the external environment on saliva samples, we only selected saliva samples of children living in the same area. We investigated the microbial communities in saliva samples from 5-year-old children with versus without dental caries and provided evidence for the diagnosis and prevention of dental caries in 5-year-old children from the perspective of microbiology. However, the sample size is slightly small. Moreover, saliva samples are affected by various factors, such as environmental factors and dietary factors, resulting in inconsistencies between the results of this study and other research results. Therefore, it is necessary to increase the sample size to further discuss and validate the results of this study.

## Conclusion

In conclusion, alpha diversity analysis demonstrated that the richness and diversity of the bacterial communities were similar between high caries and no caries children. Then, PCoA and hierarchical clustering showed that the two groups had similar microorganisms. Meanwhile, LEfSe analysis detected several bacteria at significantly higher levels in the high caries group, which can be recognized as a potential bacterial biomarker. A portion of the detected microorganisms was shared in the two groups, supporting the existence of an oral core microbiome. These bacteria play a critical role in maintaining the balance of the oral microbial ecosystem. KEGG maps showed differences between the two groups, which may indicate that the function of the microbiota was to maintain the balance of the oral microbial ecosystem. Parental education and counseling on the importance of a healthy microbiome and diet in infancy should be conducted as early as possible.

## Supplementary Information

**Additional file 1:** **Appendix 1.** A total of 10980 operational taxonomic unitsidentified by analysis of 97 percent of qualified sequences.

## Data Availability

The data of this study are available from the corresponding author upon reasonable request.

## Abbreviations

OTUs: Operational taxonomic units
PCoA: Principal coordinate analysis
ANOSIM: Analysis of similarities
LEfSe: Linear discriminant analysis of effect size
PICRUSt2: Phylogenetic investigation of communities by reconstruction of unobserved states
KEGG: Kyoto encyclopedia of genes and genomes

## References

[1] Wang X (2018). The 4th National Oral Health Survey in the Mainland of China.

[2] Marsh PD (1994). Microbial ecology of dental plaque and its significance in health and disease. Adv Dent Res.

[3] Marsh PD (2006). Dental diseases--are these examples of ecological catastrophes?. Int J Dent Hyg.

[4] Takahashi N, Nyvad B (2008). Caries ecology revisited: microbial dynamics and the caries process. Caries Res.

[5] World Health Organization (2013). Oral Health Surveys: Basic Methods.

[6] Jiang Q, Liu J, Chen L, Gan N, Yang D (2019). The oral microbiome in the elderly with dental caries and health. Front Cell Infect Microbiol.

[7] Robert Edgar C (2013). UPARSE: highly accurate OTU sequences from microbial amplicon reads.[J]. Nat Methods.

[8] Bolger AM, Lohse M, Usadel B (2014). Trimmomatic: a flexible trimmer for Illumina sequence data. Bioinformatics.

[9] Edgar RC, Haas BJ, Clemente JC, Quince C, Knight R (2011). UCHIME improves sensitivity and speed of chimera detection. Bioinformatics.

[10] Wang Q, Garrity GM, Tiedje JM, Cole JR (2007). Naive Bayesian classifier for rapid assignment of rRNA sequences into the new bacterial taxonomy. Appl Environ Microbiol.

[11] Quast C, Pruesse E, Yilmaz P, et al. The SILVA ribosomal RNA gene database project: improved data processing and web-based tools. Nucleic Acids Res. 2013;41(Database issue):D590-6. 10.1093/nar/gks1219 PMC3531112 23193283

[12] Schloss PD, Westcott SL, Ryabin T (2009). Introducing mothur: open-source, platform-independent, community-supported software for describing and comparing microbial communities. Appl Environ Microbiol.

[13] Lozupone CA, Hamady M, Kelley ST, Knight R (2007). Quantitative and qualitative beta diversity measures lead to different insights into factors that structure microbial communities. Appl Environ Microbiol.

[14] Segata N, Izard J, Waldron L, Gevers D, Miropolsky L, Garrett WS, Huttenhower C (2011). Metagenomic biomarker discovery and explanation. Genome Biol.

[15] Kanehisa M, Furumichi M, Sato Y, Kawashima M, Ishiguro-Watanabe M (2023). KEGG for taxonomy-based analysis of pathways and genomes. Nucleic Acids Res.

[16] Ward T, Larson J, Meulemans J, et al. BugBase predicts organism level microbiome phenotypes. bioRxiv. 2017.

[17] Chen H, Jiang W (2014). Application of high-throughput sequencing in understanding human oral microbiome related with health and disease. Front Microbiol.

[18] Chen T, Shi Y, Wang X (2017). High-throughput sequencing analyses of oral microbial diversity in healthy people and patients with dental caries and periodontal disease. Mol Med Rep.

[19] Fábián TK, Fejérdy P, Csermely P (2008). Salivary genomics, transcriptomics and proteomics: the emerging concept of the oral ecosystem and their use in the early diagnosis of cancer and other diseases. Curr Genomics.

[20] Jiang W, Ling Z, Lin X (2014). Pyrosequencing analysis of oral microbiota shifting in various caries states in childhood. Microb Ecol.

[21] Gross EL, Leys EJ, Gasparovich SR (2010). Bacterial 16S sequence analysis of severe caries in young permanent teeth. J Clin Microbiol.

[22] Zhang M, Chen Y, Xie L (2015). Pyrosequencing of plaque microflora in twin children with discordant caries phenotypes. PLoS ONE.

[23] Zhang WT. Macrogenomic study on oral microbial community in 3–5-year-old children with different caries sensitivity in three ethnic groups in Bozhou region. Xinjiang Medical University, 2019.

[24] Zhao MJ (2021). Preliminary study on the relationship between the structural changes of intestinal flora and the occurrence and development of dental caries. Zunyi Medical University.

[25] Teng F, Yang F, Huang S (2015). Prediction of early childhood caries via spatial-temporal variations of oral microbiota. Cell Host Microbe.

[26] He J, Tu Q, Ge Y (2018). Taxonomic and functional analyses of the supragingival microbiome from caries-affected and no caries hosts. Microb Ecol.

[27] Ling Z, Liu X, Luo Y (2013). Pyrosequencing analysis of the human microbiota of healthy Chinese undergraduates. BMC Genomics.

[28] Xiao X, Li Y, Xiao L (2013). The novel species and genus discovered and nominated from the human oral cavity in 2009–2012. West China J Stomatol.

[29] Agnello M, Marques J, Cen L (2017). Microbiome associated with severe caries in canadian first nations children. J Dent Res.

[30] Johansson I, Witkowska E, Kaveh B (2016). The microbiome in populations with a low and high prevalence of caries. J Dent Res.

[31] Tanner AR, Kressirer CA, Faller LL (2016). Understanding caries from the oral microbiome perspective. J Calif Dent Assoc.

[32] Kassebaum NJ, Smith AGC, Bernabé E (2017). Global, regional, and national prevalence, incidence, and disability-adjusted life years for oral conditions for 195 countries, 1990–2015: a systematic analysis for the global burden of diseases, injuries, and risk factors. J Dent Res.

[33] Marsh PD, Zaura E (2017). Dental biofilm: ecological interactions in health and disease. J Clin Periodontol.

[34] Wang Y, Wang S, Wu C (2019). Oral microbiome alterations associated with early childhood caries highlight the importance of carbohydrate metabolic activities. mSystems.

[35] Liu G, Wu C, Abrams WR, Li Y (2020). Structural and functional characteristics of the microbiome in deep-dentin caries. J Dent Res.

[36] Milnes AR, Bowden GH (1985). The microflora associated with developing lesions of nursing caries. Caries Res.

[37] Van Houte J, Jordan HV, Laraway R (1990). Association of the microbial flora of dental plaque and saliva with human root-surface caries. J Dent Res.

[38] Ling Z, Kong J, Jia P (2010). Analysis of oral microbiota in children with dental caries by PCR-DGGE and barcoded pyrosequencing. Microb Ecol.

[39] Xu H, Hao W, Zhou Q (2014). Plaque bacterial microbiome diversity in children younger than 30 months with or without caries prior to eruption of second primary molars. PLoS ONE.

[40] Jiang S, Gao X, Jin L, Lo EC (2016). Salivary microbiome diversity in no caries and caries-affected children. Int J Mol Sci.

[41] Parahitiyawa NB, Scully C, Leung WK (2010). Exploring the oral bacterial flora: current status and future directions. Oral Dis.

[42] Kirst ME, Li EC, Alfant B (2015). Dysbiosis and alterations in predicted functions of the subgingival microbiome in chronic periodontitis. Appl Environ Microbiol.

[43] Shimada A, Noda M, Matoba Y (2015). Oral lactic acid bacteria related to the occurrence and/or progression of dental caries in Japanese preschool children. Biosci Microbiota Food Health.

[44] Xu L, Chen X, Wang Y (2018). Dynamic alterations in salivary microbiota related to dental caries and age in preschool children with deciduous dentition: a 2-year follow-up study. Front Physiol.

